# The lauric acid-activated signaling prompts apoptosis in cancer cells

**DOI:** 10.1038/cddiscovery.2017.63

**Published:** 2017-09-18

**Authors:** Rosamaria Lappano, Anna Sebastiani, Francesca Cirillo, Damiano Cosimo Rigiracciolo, Giulia Raffaella Galli, Rosita Curcio, Roberta Malaguarnera, Antonino Belfiore, Anna Rita Cappello, Marcello Maggiolini

**Affiliations:** 1 Department of Pharmacy, Health and Nutritional Sciences, University of Calabria, Rende, Italy; 2 Department of Health Sciences, University Magna Graecia of Catanzaro, Catanzaro, Italy

## Abstract

The saturated medium-chain fatty-acid lauric acid (LA) has been associated to certain health-promoting benefits of coconut oil intake, including the improvement of the quality of life in breast cancer patients during chemotherapy. As it concerns the potential to hamper tumor growth, LA was shown to elicit inhibitory effects only in colon cancer cells. Here, we provide novel insights regarding the molecular mechanisms through which LA triggers antiproliferative and pro-apoptotic effects in both breast and endometrial cancer cells. In particular, our results demonstrate that LA increases reactive oxygen species levels, stimulates the phosphorylation of EGFR, ERK and c-Jun and induces the expression of c-fos. In addition, our data evidence that LA via the Rho-associated kinase-mediated pathway promotes stress fiber formation, which exerts a main role in the morphological changes associated with apoptotic cell death. Next, we found that the increase of p21^Cip1/WAF1^ expression, which occurs upon LA exposure in a p53-independent manner, is involved in the apoptotic effects prompted by LA in both breast and endometrial cancer cells. Collectively, our findings may pave the way to better understand the anticancer action of LA, although additional studies are warranted to further corroborate its usefulness in more comprehensive therapeutic approaches.

## Introduction

Fatty acids are acyclic carboxylic acids with aliphatic tails of different lengths. Based on their carbon atom chain length, fatty acids are classified into the following three groups: short-chain fatty acids with <6 carbon atoms, medium-chain fatty acids (MCFA) and long-chain fatty acids that contain 6–12 carbons and >12 carbons, respectively.^1^ Fatty acids are major components of triacylglycerols, phospholipids and other complex lipids, therefore representing main contributors to dietary fat in humans.^2^ Plant oils like palm, coconut and olive oils, nuts, seeds and seed oils, cocoa butter and animal-derived fats as lard, tallow and butter, are rich of fatty acids that are important components of cell membranes and essential sources of energy.^2^ Previous studies have demonstrated that fatty acids are also involved in diverse transduction pathways, in gene transcription and relevant biological events as cell metabolism, inflammation, apoptosis and production of bioactive lipid mediators, thus contributing to multiple patho-physiological responses.^2–7^

Lauric acid (LA), which is a saturated MCFA with 12 carbon atoms and the primary fatty acid of coconut oil, has been associated with certain health benefits of coconut oil intake.^8–10^ LA is also contained in plant oils, fruits, seeds and in breast milk.^11,12^ LA has been shown to elicit diverse actions in various tissues, including a potent antimicrobial property.^8^ For instance, LA and the derivative monolaurin were reported to destroy cell membranes of gram-positive bacteria and lipid-coated viruses, to interfere with main cellular responses as the activation of transduction cascades and gene transcription, to stabilize cell membranes toward the prevention of bacterial resistance.^8^ In addition, LA promoted inflammatory processes activating the nuclear factor-*κ*B transcription factor as well as stimulating the expression of cyclooxygenase-2 and pro-inflammatory cytokines.^13^ LA was also associated with beneficial effects on the cardiovascular system due to its ability to increase the high-density lipoproteins^14^ and to reduce the blood pressure and heart rate in both normotensive and hypertensive rats.^15^ Moreover, LA prevented the prostatic hyperplasia induced by testosterone in rats,^16^ triggered apoptosis in colon cancer cells through oxidative stress^17^ and improved the sensitization of the EGFR inhibitor cetuximab in KRAS/BRAF mutated colorectal cancer cells.^18^ It is worth mentioning that the consumption of virgin coconut oil during chemotherapy improved the global quality of life in patients with breast cancer.^19^

Here, we show for the first time that LA elicits antiproliferative and pro-apoptotic effects in breast and endometrial cancer cells promoting the generation of reactive oxygen species (ROS), the activation of transduction pathways and gene expression changes. In particular, the upregulation of the cyclin-dependent kinase inhibitor p21^Cip1/WAF1^ upon LA exposure was found to be required for its anticancer properties. Our findings shed new light on the molecular mechanisms through which LA induces antiproliferative and pro-apoptotic responses in both breast and endometrial cancer cells toward its usefulness in more comprehensive therapeutic approaches.

## Results

### LA inhibits cancer cell viability

**Figure 1:**
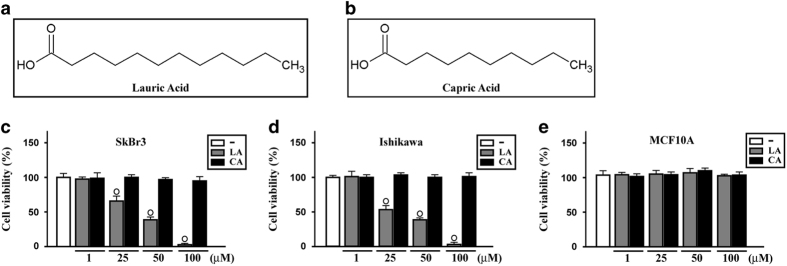
Lauric acid inhibits the proliferation of breast and endometrial cancer cells. (**a**, **b**) Chemical structures of lauric acid (LA) and capric acid (CA). (**b**–**d**) MTT growth assays in SkBr3 (**c**), Ishikawa (**d**) and MCF-10A (**e**) cells treated for 48 h with vehicle (−) or increasing concentrations of LA and CA, as indicated. Cell viability is expressed as the percentage of cells upon treatments respect to cells treated with vehicle. Values shown are mean±S.D. of three independent experiments performed in triplicate. (○) indicates *P*<0.05 for cells receiving vehicle versus treatments.

On the basis of previous findings showing that MCFAs may elicit apoptosis in certain cancer cells^17,20^ and considering that in our recent investigation LA exerted antiproliferative activity in diverse types of tumor cells,^21^ we began the present study evaluating whether LA (Figure 1a) and a further MCFA namely capric acid (CA) (Figure 1b) may affect the viability of SkBr3 breast and Ishikawa endometrial cancer cells, which were used as model system. Only LA inhibited the viability of both cancer cell types (Figures 1c and d) without altering the growth of MCF-10A normal breast epithelial cells (Figure 1e), thus suggesting its specific potential to trigger antiproliferative effects in malignant cells.

### LA triggers ROS generation and EGFR, ERK and c-Jun phosphorylation

**Figure 2:**
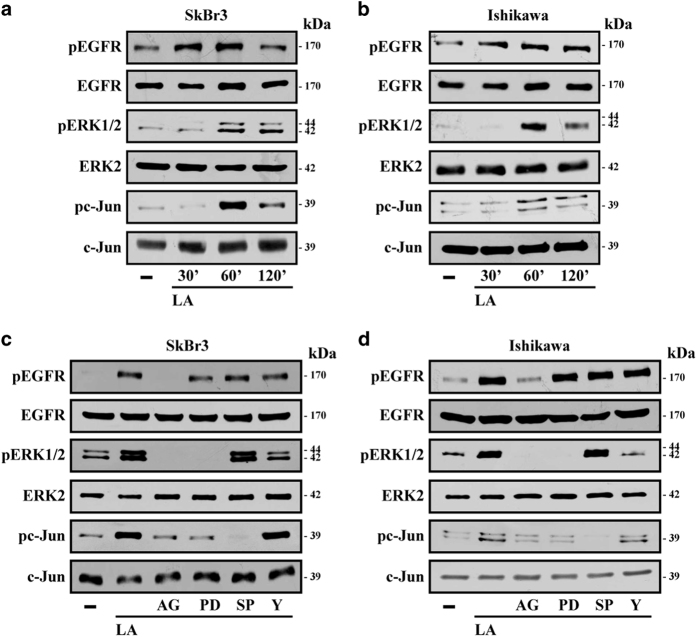
Lauric acid triggers rapid responses in breast and endometrial cancer cells. (**a**, **b**) Phosphorylation of EGFR, ERK1/2 and c-Jun in SkBr3 (**a**) and Ishikawa (**b**) cells treated with vehicle (−) and 100 *μ*M LA, as indicated. (**c**,** d**) EGFR, ERK1/2 and c-Jun activation in SkBr3 (**c**) and Ishikawa (**d**) cells treated for 60 min with vehicle or 100 *μ*M LA alone or in combination with 10 *μ*M EGFR inhibitor AG1478 (AG), 10 *μ*M MEK inhibitor PD98089 (PD), 1 *μ*M JNK inhibitor SP 600125 (SP) and 10 *μ*M ROCK inhibitor Y-27632 (Y). EGFR, ERK2 and c-Jun were used as loading controls for pEGFR, pERK1/2 and pc-Jun, respectively. Results shown are representative of at least two independent experiments.

**Figure 3:**
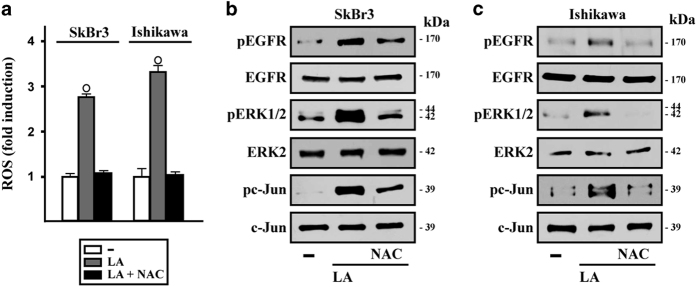
ROS generation by lauric acid is involved in the activation of transduction signaling observed in breast and endometrial cancer cells. (**a**) ROS production determined as DCF fluorescence in SkBr3 and Ishikawa cells treated for 60 min with vehicle (−) or 100 *μ*M LA alone or in combination with 300 *μ*M free radical scavenger NAC. DCF fluorescence obtained in cells treated with vehicle was set as onefold induction upon which ROS levels induced by treatments were calculated. Data shown are the mean±S.D. of three independent experiments performed in triplicate. (○) indicates *P*<0.05 for cells receiving vehicle versus treatments. EGFR, ERK1/2 and c-Jun activation in SkBr3 (**b**) and Ishikawa (**c**) cells treated for 60 min with vehicle or 100 *μ*M LA alone or in combination with 300 *μ*M NAC. EGFR, ERK2 and c-Jun were used as loading controls for pEGFR, pERK1/2 and pc-Jun, respectively. Results shown are representative of at least two independent experiments.

To evaluate the molecular mechanisms involved in the ability of LA to lower cancer cell viability, we ascertained that LA triggers the phosphorylation of EGFR, ERK and c-Jun in both SkBr3 and Ishikawa cells (Figures 2a and b). These responses were no longer observed in the presence of the EGFR inhibitor (AG) (Figures 2c and d), whereas ERK activation by LA was abolished using the MEK inhibitor (PD) and the Rho-associated kinase (ROCK) inhibitor (Y) but it still persisted using the JNK inhibitor (SP) (Figures 2c and d). The phosphorylation of c-Jun by LA was prevented in the presence of PD or SP, but not using the ROCK inhibitor Y (Figures 2c and d). Reminiscing previous data on the ability of LA to induce ROS levels in colon cancer cells,^17^ we found that LA triggers ROS generation in our model system, yet this response was no longer evident using the ROS scavenger *N*-acetyl-l-cysteine (NAC, Figure 3a). Thereafter, we established that the phosphorylation of EGFR, ERK and c-Jun upon LA exposure is strictly dependent on ROS generation, as ascertained using NAC in both cell types (Figures 3b and c).

### LA induces gene expression changes

**Figure 4:**
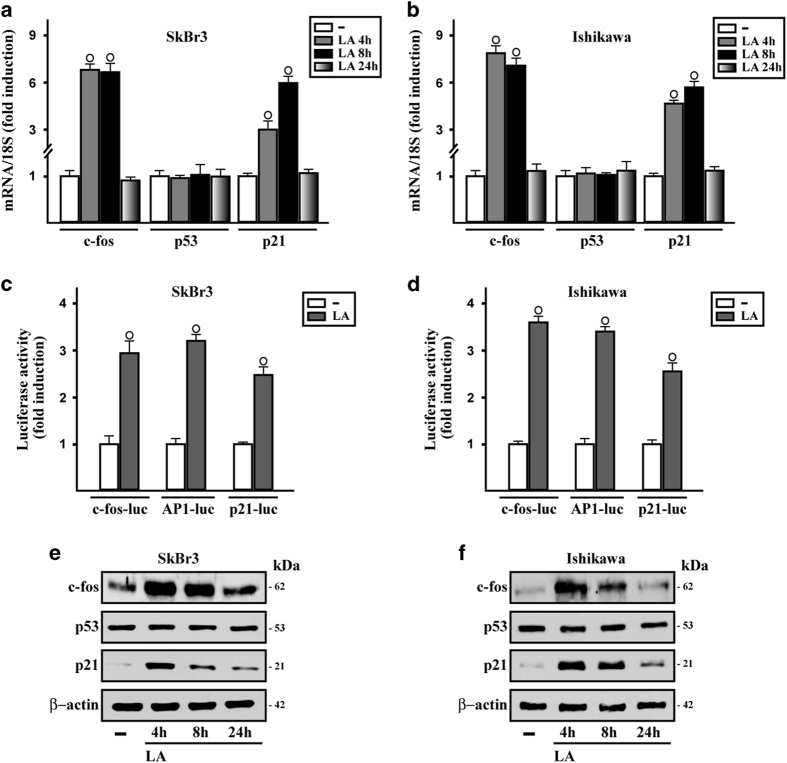
Lauric acid regulates the expression of cell cycle regulatory genes. The mRNA expression of c-fos, p53 and p21^Cip1/WAF1^ (p21) was evaluated by real-time PCR in SkBr3 (**a**) and Ishikawa (**b**) cells treated with vehicle (−) or 100 *μ*M LA, as indicated. Data obtained from three independent experiments performed in triplicate were normalized to 18 S expression and shown as fold changes of mRNA expression induced by LA respect to cells treated with vehicle. Evaluation of c-fos, AP1 and p21 luciferase reporter genes in SkBr3 (**c**) and Ishikawa (**d**) cells treated for 18 h with vehicle or 100 *μ*M LA. The luciferase activities were normalized to the internal transfection control, and values of cells receiving vehicle were set as onefold induction upon which the activity induced by treatments was calculated. Data shown are the mean±S.D. of three independent experiments performed in triplicate. (○) indicates *P*<0.05 for cells receiving vehicle versus treatments. c-fos, p53 and p21 protein levels in SkBr3 (**e**) and Ishikawa (**f**) cells treated with vehicle or 100 *μ*M LA, as indicated. *β*-actin was used as a loading control. Results shown are representative of at least two independent experiments.

**Figure 5:**
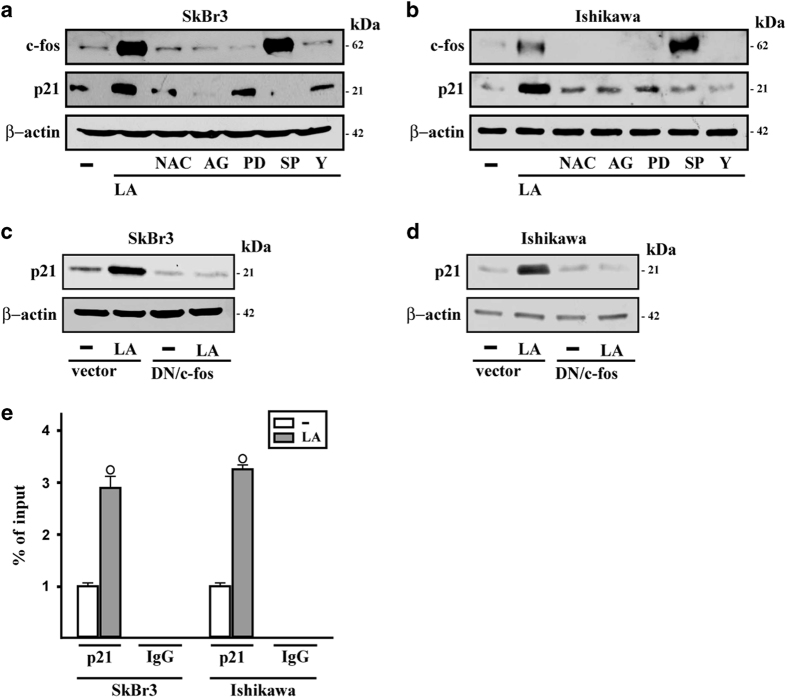
c-fos is involved in the upregulation of p21^Cip1/WAF1^ induced by lauric acid. Immunoblots of c-fos and p21^Cip1/WAF1^ (p21) in SkBr3 (**a**) and Ishikawa (**b**) cells treated for 4 h with vehicle (−) or 100 *μ*M LA alone or in combination with 300 *μ*M free radical scavenger NAC, 10 *μ*M EGFR inhibitor AG1478 (AG), 10 *μ*M MEK inhibitor PD98089 (PD), 1 *μ*M JNK inhibitor SP 600125 (SP) and 10 *μ*M ROCK inhibitor Y-27632 (Y). The expression vector encoding for a dominant-negative form of c-fos (DN/c-fos) blocked the upregulation of p21^Cip1/WAF1^ protein levels induced by 100 *μ*M LA in SkBr3 (**c**) and Ishikawa (**d**) cells. *β*-actin was used as a loading control. Results shown are representative of at least two independent experiments. (**e**) Recruitment of c-fos induced by 100 *μ*M LA to the AP1 site located within the p21^Cip1/WAF1^ promoter sequence in SkBr3 and Ishikawa cells, as indicated. In control samples non-specific IgG was used instead of the primary antibody. Each column represents the mean±S.D. of three independent experiments. (○) indicates *P*<0.05 for cells receiving vehicle versus treatments.

Then, we assessed the expression levels of well-known cell cycle regulators as the member of the AP1 transcription factor complex namely c-fos, the tumor suppressor p53 and the cyclin-dependent kinase inhibitor p21^Cip1/WAF1^. In both SkBr3 and Ishikawa cells, LA upregulated the mRNA expression of c-fos and p21^Cip1/WAF1^, without altering the levels of p53 (Figures 4a and b). In addition, LA transactivated the AP1-luc responsive collagenase promoter construct that was transiently transfected in SkBr3 and Ishikawa cells and stimulated the transcriptional activity of reporter plasmids containing the c-fos and p21^Cip1/WAF1^ promoter sequences (Figures 4c and d). According to the results obtained in real-time PCR, LA did not modify the p53 protein levels, whereas it increased c-fos and p21^Cip1/WAF1^ protein expression in both cell types (Figures 4e and f). We next ascertained that the ROS scavenger NAC, the EGFR inhibitor AG, the MEK inhibitor PD and the ROCK inhibitor Y prevent c-fos induction by LA (Figures 5a and b). Likewise, these compounds together with the JNK inhibitor SP repressed the increase of p21^Cip1/WAF1^ protein levels elicited by LA (Figures 5a and b). As the upregulation of p21^Cip1/WAF1^ protein levels was no longer evident transfecting a dominant-negative form of c-fos (DN/c-fos) in both SkBr3 and Ishikawa cells (Figures 5c and d), we ascertained by chromatin immunoprecipitation assay that LA induces the recruitment of c-fos to the AP1 site located within the p21^Cip1/WAF1^ promoter sequence (Figure 5e). Overall, these data indicate that c-fos-AP1 transduction signaling is involved in the upregulation of p21^Cip1/WAF1^ induced by LA.

### LA promotes stress fiber formation and apoptosis in cancer cells

**Figure 6:**
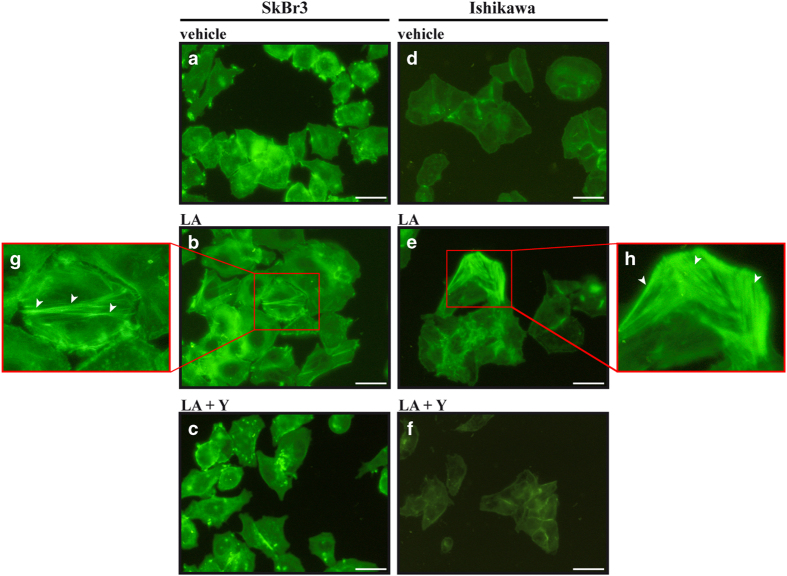
Lauric acid promotes the formation of stress fibers. SkBr3 and Ishikawa cells were treated for 4 h with vehicle (−) (**a**,** d**) or 100 *μ*M LA alone (**b**, **e**) or in combination with 10 *μ*M ROCK inhibitor Y-27632 (Y) (**c**,** f**) and subjected to phalloidin staining to visualize F-actin. (**g**,** h**) Enlarged details of stress fibers shown in** b** and** e**, respectively. White arrows indicate stress fibers. Images shown are representative of 30 random fields obtained in three independent experiments. Scale bar: 12.5 *μ*m.

**Figure 7:**
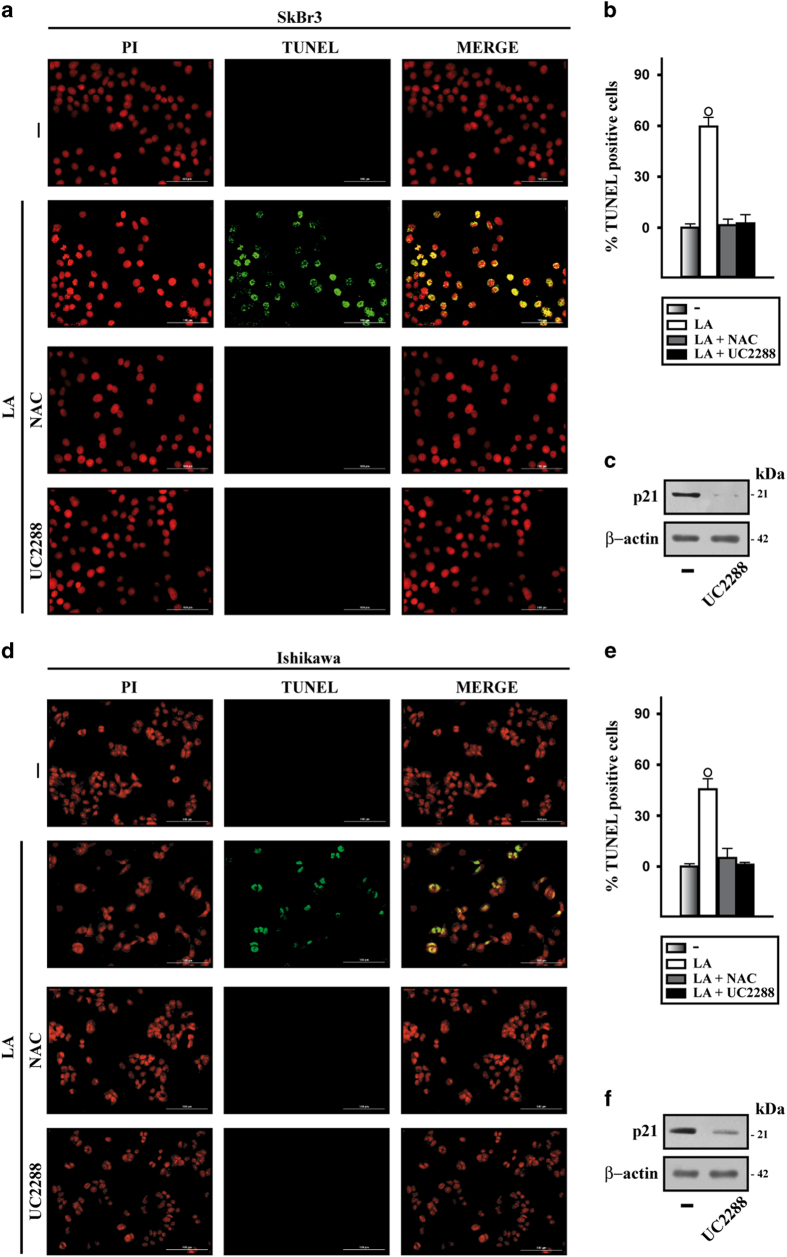
Lauric acid induces apoptotic cell death. (**a**,** d**) TdT-mediated dUTP nick-end-labeling (TUNEL) staining (green) in SkBr3 (**a**) and Ishikawa (**d**) cells treated for 18 h with vehicle (−) or 100 *μ*M LA alone or in combination with 300 *μ*M free radical scavenger NAC and 10 *μ*M p21^Cip1/WAF1^ inhibitor UC2288, as indicated. Nuclei were stained by propidium iodide (PI, red). Magnification is indicated by bars (100 *μ*m). Each experiment shown is representative of 20 random fields observed. (**b**, **e**) Bar graphs represent the percentage of TUNEL-positive cells upon treatments versus vehicle. Values are the mean of three independent experiments. (○) indicates *P*<0.05 for cells receiving vehicle versus treatments. (**c**,** f**) Efficacy of p21^Cip1/WAF1^ downregulation by UC2288. *β*-actin was used as a loading control. Results shown are representative of at least two independent experiments.

Rho GTPases and their effectors as the Rho-associated protein kinase (ROCK) are key regulators of the cytoskeleton reorganization and the generation of the contractile force required for stress fiber formation.^22^ In line with the aforementioned findings regarding the capability of the ROCK inhibitor to prevent LA-induced responses, in both SkBr3 and Ishikawa cells LA promoted the formation of stress fibers in a ROCK-dependent manner as this effect was abrogated in the presence of its inhibitor (Figures 6a-f) that alone did not show any effects (data not shown). Then, we assessed that LA increases the percentage of SkBr3 (Figures 7a–c) and Ishikawa (Figures 7d-f) TdT-mediated dUTP nick-end-labeling (TUNEL)-positive cells, however this effect was prevented in the presence of the ROS scavenger NAC (Figure 7). In addition, the apoptotic effects induced by LA were blocked in the presence of the p21^Cip1/WAF1^ inhibitor UC2288 (Figure 7), suggesting that p21^Cip1/WAF1^is involved in the pro-apoptotic activity exerted by LA in breast and endometrial cancer cells.

## Discussion

The present study provides novel evidence regarding the molecular mechanisms through which LA elicits antiproliferative and pro-apoptotic effects in breast and endometrial cancer cells. In particular, we have ascertained that ROS generation induced by LA triggers the activation of the EGFR/ERK/AP1 transduction pathway, leading to the upregulation of p21^Cip/WAF1^ in a p53-independent manner.

Fatty acids are structural components of cellular membranes either alone or together with other molecules as phospholipids and triacylglycerides.^23^ In addition, fatty-acid oxidation occurring at the mitochondrial level plays a pivotal role in maintaining energy homeostasis during catabolic states.^24^ Nevertheless, fatty acids are currently no longer considered as mere membrane structure regulators or energy sources as they also influence diverse transduction signaling and cellular functions.^2–7^ For instance, regulating transcription factors involved in lipid metabolism and inflammation, saturated fatty acids as LA may influence the biosynthesis of cholesterol and triacylglycerols, the assembly, secretion and clearance of lipoproteins, various metabolic and inflammatory processes.^23^ Therefore, an increasing attention has been paid to the multifaceted role elicited by fatty acids on human health given that the amount and type of fatty acids contained in the diet are involved in the etiopathogenesis of diabetes, cancer and cardiovascular, immunity, inflammatory, renal, hepatic diseases.^25^ In this context, coconut oil that is one of the richest sources of saturated fatty acids as LA, has attracted interest for its potential health benefits.^26–29^ Furthermore, coconut oil has been shown to counteract the action of stimulatory agents in colon and mammary tumors in rats^30,31^ and to improve the quality of life of breast cancer patients undergoing chemotherapy.^19^ As it concerns LA, Fauser and co-workers^17^ firstly demonstrated its ability to induce apoptosis in colon cancer cells through the reduction of glutathione levels and the generation of oxidative stress. In accordance with these and other observations showing that fatty acids may induce ROS generation in diverse types of cells,^32–34^ we have extended these findings ascertaining that LA prompts ROS-mediated apoptosis also in breast and endometrial cancer cells through the subsequent activation of relevant transduction pathways. In this respect, it is worth mentioning that the EGFR and ERK signaling are mostly referred to as regulatory pathways of cell proliferation, migration and differentiation.^35,36^ Nevertheless, these two main transduction mediators can also trigger apoptotic signals especially in the context of tumor cells.^35,36^ For instance, EGF through the cognate receptor induced the expression of the caspase 1 enzyme and p21^WAF1/CIP1^ toward apoptosis and growth inhibition.^35^ In addition, it has been demonstrated that free radicals generated by radiation exposure may elicit the activation of the EGFR/ERK signaling in cancer cells.^36^ In line with these observations, we found that LA increases ROS levels that in turn trigger the EGFR/ERK transduction pathway and gene expression changes, therefore culminating in apoptotic responses in cancer cells.

Actin stress fibers have a pivotal role in many cellular functions, including cell adhesion, mobility, contraction and morphogenesis.^37^ Stress fibers are also required for membrane blebbing, nuclear disintegration and apoptosis.^38–41^ The small GTPase Rho and its main effector ROCK are involved in several cellular processes like the regulation of actin cytoskeleton, cell polarity, microtubule dynamics, gene transcription, cell cycle progression, differentiation, apoptosis and the formation of actin stress fibers.^37,42–44^ In this vein, ROCK was shown to mediate the generation of stress fibers that in turn trigger the p21^Cip1/WAF1^-dependent apoptosis upon phorbol 12-myristate 13-acetate exposure in prostate cancer cells.^40^ Further extending these data, our findings have determined for the first time that LA promotes in breast and endometrial cancer cells the formation of stress fibers through the ROCK transduction pathway, thus suggesting that LA might be included among the activators of the Rho/ROCK signaling.

The cyclin-dependent kinase inhibitor p21^Cip1/WAF1^ has an essential role in the cell cycle arrest, the transcriptional regulation, the inhibition of DNA replication, the DNA repair, the stress-induced premature senescence and the modulation of apoptosis.^45–48^ Numerous studies have shown that p21^Cip1/WAF1^ can mediate both pro- and anti-apoptotic functions depending on the type of stimulation and the cellular context.^48^ For instance, p21^Cip1/WAF1^ can prevent cells from undergoing apoptosis triggering cell cycle arrest, inactivating cyclin A/Cdk2 complexes, inhibiting the activity of procaspase 3, caspase 8 and 10, stress-activated protein kinases and apoptosis signal-regulating kinase 1.^47,49^ Likewise, several reports have also suggested that p21^Cip1/WAF1^ exerts a pro-apoptotic function under certain cellular stresses upregulating the pro-apoptotic protein Bax, activating the tumor necrosis factor family of death receptors and regulating components of the DNA repair machinery.^47^ It is worth mentioning that even though p21^Cip1/WAF1^ may represent a major p53 transcriptional target, it can promote apoptosis through both p53-dependent and independent mechanisms.^47^ In addition, p21^Cip1/WAF1^ can act as a tumor suppressor or an oncogene depending on the stimulations and the cellular context.^45^ In particular, various compounds eliciting an anticancer activity such as histone deacetylase inhibitors, cisplatin, phorbol 12-myristate 13-acetate and curcumin were shown to induce apoptotic cell death through the p21^Cip1/WAF1^ induction.^40,50–54^ Extending these findings, our data have ascertained that LA induces apoptosis in both breast and endometrial cancer cells upregulating the p21^Cip1/WAF1^ expression levels via an AP1-mediated pathway.

Overall, the present results provide novel insights into the potential of LA to activate the EGFR/ERK/AP1/p21^Cip1/WAF1^ transduction signaling toward antiproliferative and pro-apoptotic responses in tumor cells. Nevertheless, further experimental evidence are warranted to better define the action of LA alone or in the context of coconut oil consumption on tumor development as claimed by a current newsworthy debate.

## Materials and methods

### Reagents

LA, CA, NAC, Y-27632 (Y) and 2′,7′-dichlorofluorescin diacetate (DCFDA) were purchased from Sigma-Aldrich (Milan, Italy). Tyrphostin AG1478 (AG), PD98059 (PD), SP 600125 (SP) and UC2288 were obtained from Calbiochem (DBA, Milan, Italy). All compounds were dissolved in dimethyl sulfoxide except LA, CA, NAC and Y-27632 (Y), which were solubilized in water.

### Cell cultures

SkBr3 breast cancer cells were obtained by ATCC, used <6 months after resuscitation and maintained in RPMI 1640 without phenol red supplemented with 10% FBS and 100 mg/ml penicillin/streptomycin (Life Technologies, Milan, Italy). Ishikawa endometrial cancer cells were obtained by D Picard (University of Geneva) and maintained in MEM supplemented with 10% FBS, 100 *μ*g/ml penicillin/streptomycin, 2 mM L-glutamine and 1% Non-Essential Amino Acids Solution Cells (Life Technologies). Cells were switched to medium without serum the day before immunoblots and reverse transcription-PCR experiments.

### Cell viability assay

Cell viability was determined by the MTT [3-(4,5-dimethylthiazol-2-yl)-2,5-diphenyltetrazolium bromide] assay, which is based on the conversion of MTT to MTT formazan by mitochondrial enzyme. Cells were seeded in quadruplicate in 96-well plates in regular growth medium and grown until 70–80% confluence. Cells were washed once they had attached and then treated with increasing concentrations of each compound for 48 h in regular medium supplemented with 1% FBS. Relative cell viability was determined by MTT assay according to the manufacturer’s protocol (Sigma-Aldrich). Mean absorbance of cells receiving vehicle (−) was set as onefold induction upon which the mean absorbance of treatments was calculated.

### Plasmids, transfections and gene reporter assays

The luciferase reporter plasmid for c-fos encoding a 2.2-kb 5´ upstream fragment of human c-fos was a gift from Dr. K Nose (Hatanodai, Shinagawa-ku, Tokyo). The luciferase reporter plasmid for AP1 responsive collagen promoter was a kind gift from H Van Dam (Department of Molecular Cell Biology, Leiden University, Leiden, Netherlands). The human p21^Cip1/WAF1^ promoter-luciferase reporter was kindly provided by Dr T Sakai (Kyoto Prefectural University of Medicine, Kyoto, Japan). The *Renilla* luciferase expression vector pRL-TK (Promega, Milan, Italy) was used as internal transfection control in luciferase assays. Cells (1×10^5^) were plated into 24-well plates with regular growth medium/well the day before transfection. Cell medium was replaced on the day of transfection with serum-free medium and transfection was performed using X-tremeGENE 9 DNA Transfection Reagent (Sigma-Aldrich) and a mixture containing 0.5 *μ*g of each reporter plasmid and 5 ng of pRL-TK. After 6 h, treatments were added and cells were incubated for 18 h. Luciferase activity was measured using the Dual Luciferase Kit (Promega) according to the manufacturer’s recommendations. Firefly luciferase activity was normalized to the internal transfection control provided by the *Renilla* luciferase activity. Normalized relative light unit values obtained from cells treated with vehicle were set as onefold induction upon which the activity induced by treatments was calculated.

The plasmid DN/c-fos, which encodes a c-fos mutant that heterodimerizes with c-fos dimerization partners but does not allow DNA biding,^55^ was a kind gift from Dr C Vinson (NIH, Bethesda, MD, USA). Cells were plated onto 10-cm dishes and prior to treatments cells were transfected for 24 h using X-tremeGENE 9 DNA Transfection Reagent (Sigma-Aldrich) with a control vector and the plasmid DN/c-fos.

### Gene expression studies

Total RNA was extracted and cDNA was synthesized by reverse transcription as previously described.^56^ The expression of selected genes was quantified by real-time PCR using Step One (TM) sequence detection system (Applied Biosystems Inc, Milan, Italy). Gene-specific primers were designed using Primer Express version 2.0 software (Applied Biosystems). For c-fos, p53, p21^Cip1/WAF1^ (p21) and the ribosomal protein 18S, which was used as a control gene to obtain normalized values, the primers were: 5′- CGAGCCCTTTGATGACTTCCT-3′ (c-fos forward), 5′- GGAGCGGGCTGTCTCAGA-3′ (c-fos reverse); 5′- GCTTCATGCCAGCTACTTC-3′ (p53 forward), 5′- GGCATTCTGGGAGCTTCATCT-3′ (p53 reverse); 5′- GCTTCATGCCAGCTACTTCC-3′ (p21 forward) and 5′- CTGTGCTCACTTCAGGGTCA-3′ (p21 reverse); 5′- GGCGTCCCCCAACTTCTTA-3′ (18S forward) and 5′- GGGCATCACAGACCTGTTATT-3′ (18S reverse), respectively. Assays were performed in triplicate and the results were normalized for 18S expression and then calculated as fold induction of RNA expression.

### Chromatin immunoprecipitation assay

Cells were grown in 10-cm dishes to 70–80% confluence, shifted to serum-free medium for 24 h and then treated with vehicle (−) or 100 *μ*M LA for 4 h. Thereafter, cells were cross-linked with 1% formaldehyde and sonicated. Supernatants were immunocleared with salmon DNA/protein A-agarose (Upstate Biotechnology, Inc., Lake Placid, NY, USA) and immunoprecipitated with anti c-fos (H-125) antibody or non-specific IgG (Santa Cruz Biotechnology, DBA, Milan, Italy). Pellets were washed, eluted with a buffer consisting of 1% SDS and 0.1 mol/l NaHCO3, and digested with proteinase K. DNA was obtained by phenol/chloroform extraction and precipitated with ethanol. A 4 *μ*l volume of each sample was used as template to amplify an AP1-containing region located in the p21^Cip1/WAF1^ promoter by real-time PCR. The primers used were 5′- TCAGCTGCATTGGGTAAATCCT-3′ (forward) and 5′- CTGGACACATTTCCCCACGAA-3′ (reverse). Data were normalized to the input for the immunoprecipitation.

### Western blot analysis

Cells were grown in 10-cm dishes, exposed to ligands, and then lysed as previously described.^57^ Equal amounts of whole-protein extract were resolved on a 10% SDS-polyacrylamide gel and transferred to a nitrocellulose membrane (Amersham Biosciences, Sigma-Adrich, Milan, Italy), which were probed with primary antibodies against pEGFR Tyr 1173, EGFR (1005), phosphorylated ERK1/2 (E-4), ERK2 (C-14), p-c-Jun S73, c-Jun (N), c-fos (E8), p53 (DO-1), p21 (H164) and *β*-actin (C2) (Santa Cruz Biotechnology) and then revealed using the chemiluminescent substrate for western blotting Westar Nova 2.0 (Cyanagen, Biogenerica, Catania, Italy).

### ROS production

The non-fluorescent DCFDA probe, which becomes highly fluorescent on reaction with ROS, was used to evaluate intracellular ROS production. In brief, cells (2×10^5^) were incubated with 10 *μ*M DCFDA (Sigma-Aldrich) at 37 °C for 30 min, washed with PBS and then exposed to treatments, as indicated. Cells were washed with PBS and the fluorescent intensity of DCF was measured (excitation at 485 nm and emission at 530 nm).

### Phalloidin staining

Cells were washed twice with PBS, fixed in 4% paraformaldehyde in PBS for 10 min, washed briefly with PBS, then incubated with Phalloidin-Fluorescent Conjugate (Santa Cruz Biotechnology) and visualized with the Olympus BX41 microscope and the images were taken with CSV1.14 software using a CAM XC-30 for images acquisition (Olympus Europa, Hamburg, Germany).

### TUNEL assay

Cell apoptosis was determined by TUNEL assay, conducted using DeadEnd Fluorometric TUNEL System (Promega) and performed according to the manufacturer’s instructions. In brief, cells were treated for 18 h, as indicated, then were fixed in freshly prepared 4% paraformaldehyde solution in PBS (pH 7.4) for 25 min at 4 °C. After fixation, cells were permeabilized in 0.2% Triton X-100 solution in PBS for 5 min. After washing twice with washing buffer for 5 min, the cells were covered with equilibration buffer at room temperature for 5–10 min. The labeling reaction was performed using terminal deoxynucleotidyl transferase end-labeling TdT and fluorescein-dUTP cocktail for each sample and incubated for 1 h at 37 °C where TdT catalyses the binding of fluorescein-dUTP to free 3’OH ends in the nicked DNA. After rinsing, cells were washed with 2× SSC solution buffer and subsequently incubated with propidium iodide (Sigma-Aldrich) to stain nuclei and analyzed using the Cytation 3 Cell Imaging Multimode Reader (BioTek, Winooski, VT, USA).

### Statistical analysis

Statistical analysis was done using ANOVA followed by Newman–Keuls’ testing to determine differences in means. *P*<0.05 was considered as statistically significant.

## Additional information

**Publisher’s note:** Springer Nature remains neutral with regard to jurisdictional claims in published maps and institutional affiliations.
